# Management of secondary fistulas following bariatric surgery using endoscopic vacuum-assisted closure

**DOI:** 10.1093/jscr/rjaf176

**Published:** 2025-03-28

**Authors:** Alberto Michel Macareno, Johanna Betzabe Cobos Román, Niurky De La Rosa Villa, Ariana Medina Estrada, Isaac Esparza Estrada

**Affiliations:** Obesity Not 4 Me, Department of Bariatric and Metabolic Surgery, Río Médica, Leona Vicario 1451, 3er. Piso, Zona Urbana Rio Tijuana 22010 Tijuana, B.C., México; Obesity Not 4 Me, Department of Bariatric and Metabolic Surgery, Río Médica, Leona Vicario 1451, 3er. Piso, Zona Urbana Rio Tijuana 22010 Tijuana, B.C., México; Obesity Not 4 Me, Department of Bariatric and Metabolic Surgery, Río Médica, Leona Vicario 1451, 3er. Piso, Zona Urbana Rio Tijuana 22010 Tijuana, B.C., México; Obesity Not 4 Me, Department of Bariatric and Metabolic Surgery, Río Médica, Leona Vicario 1451, 3er. Piso, Zona Urbana Rio Tijuana 22010 Tijuana, B.C., México; Obesity Not 4 Me, Department of Bariatric and Metabolic Surgery, Río Médica, Leona Vicario 1451, 3er. Piso, Zona Urbana Rio Tijuana 22010 Tijuana, B.C., México

**Keywords:** bariatric surgery, gastropleural fistula, esophagojejunal fistula, endoscopic vacuum-assisted closure (EVAC), postoperative complications

## Abstract

Obesity significantly impacts individuals’ quality of life. Bariatric surgery, including vertical sleeve gastrectomy and gastric bypass, is effective for long-term weight loss but has increased post-bariatric complications like fistulas. We treated three patients with post-bariatric complications. Cases included a gastropleural fistula secondary to vertical sleeve gastrectomy and an esophagojejunal fistula secondary to Roux-en-Y gastric bypass. Endoscopic vacuum-assisted closure (EVAC) was used, applying continuous negative pressure via a polyurethane sponge connected to suction. The EVAC technique effectively managed complex post-bariatric fistulas, facilitating closure and improving outcomes. Patient selection and technical expertise are crucial for optimizing EVAC results. This innovative approach offers a promising solution for post-bariatric complications, enhancing recovery and quality of life.

## Introduction

Obesity is a chronic disease that has reached pandemic proportions, significantly impacting individuals’ quality of life worldwide. Currently, bariatric surgery, including vertical sleeve gastrectomy and gastric bypass, is considered the most effective treatment for achieving long-term weight loss maintenance. However, the increasing number of these procedures has led to a corresponding rise in postbariatric complications [1].

Although bariatric surgery provides substantial benefits in improving patients’ quality of life, it is not without complications. One of the most severe complications is the development of leaks and fistulas, which are full-thickness disruptions secondary to the surgical techniques employed. These complications can result in significant morbidity and mortality [2].

The incidence of leaks and fistulas following bariatric surgery ranges between 1.5% and 4.9% [2]. Management of these complications involves a variety of approaches, including self-expanding prostheses, closure clips, internal pigtails, adhesive substances for endoscopic use, and gastrointestinal rest coupled with parenteral nutrition, among others [3]. Recent guidelines emphasize the importance of enhanced recovery protocols to optimize patient outcomes and reduce complications [4].

Endoscopic vacuum-assisted closure (EVAC) is a novel technique for managing fistulas. It functions by applying continuous and controlled negative pressure on the defect using a polyurethane sponge connected to a suction device. This method has proven effective in closing complex fistulas that are otherwise challenging to manage [5].

EVAC provides continuous suction and drainage of the wound, significantly reducing edema and inhibiting the growth of microorganisms. This process promotes scar tissue formation and stimulates the development of granulation tissue, contributing to effective fistula closure [6]. Studies have shown that EVAC can be a valuable tool in the armamentarium for managing postbariatric complications, offering an innovative and effective solution [7].

## Materials and methods

We describe the endoscopic treatment of three patients who underwent bariatric surgery and were referred to our center due to complications. These included a gastropleural fistula secondary to a gastric sleeve with early leakage and an esophagojejunal fistula secondary to a Roux-en-Y gastric bypass complication.

In one of the patients, the placement of the sponge was a complex process due to significant angulation of the fistula.

All patients signed written informed consent for the endoscopic management of their complications and provided consent for the anonymous publication of their medical data for academic purposes.

### Patient selection criteria for EVAC

Patients selected for EVAC therapy typically present with complex and persistent postoperative fistulas that have not responded to conventional treatments. These criteria ensure that EVAC is applied to patients who are most likely to benefit from the therapy while minimizing potential risks [6, 7]. Specific criteria include:

- Patients with postoperative fistulas such as gastropleural, gastrocutaneous, esophagocutaneous, and esophagojejunal fistulas.
- Fistulas identified and evaluated through endoscopy to determine location, size, and complexity.
- Patients without severe comorbidities that could contraindicate EVAC, such as uncontrolled cardiovascular diseases or active systemic infections.
- Patients with adequate general health to undergo multiple endoscopic procedures.
- Detailed informed consent explaining the risks, benefits, and alternatives of EVAC therapy.
- Consent for the anonymous publication of medical data for academic purposes.
- Availability for frequent follow-up and close monitoring of treatment progress.
- Ability to adhere to dietary and postoperative care recommendations during and after EVAC therapy.

## Results

### Patient A

**Figure 1:**
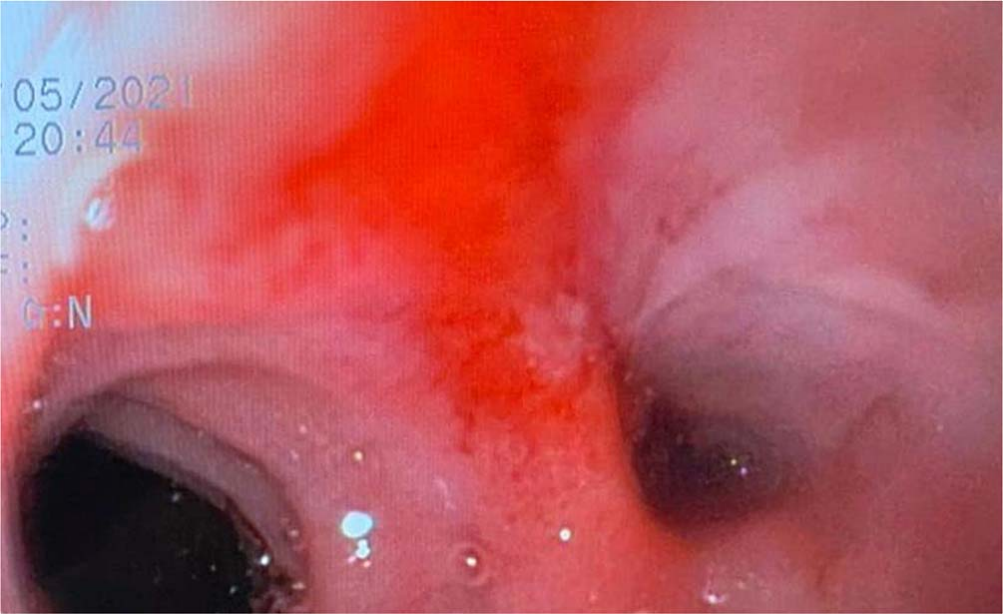
A fistulous opening is evident during the initial endoscopy.

**Figure 2:**
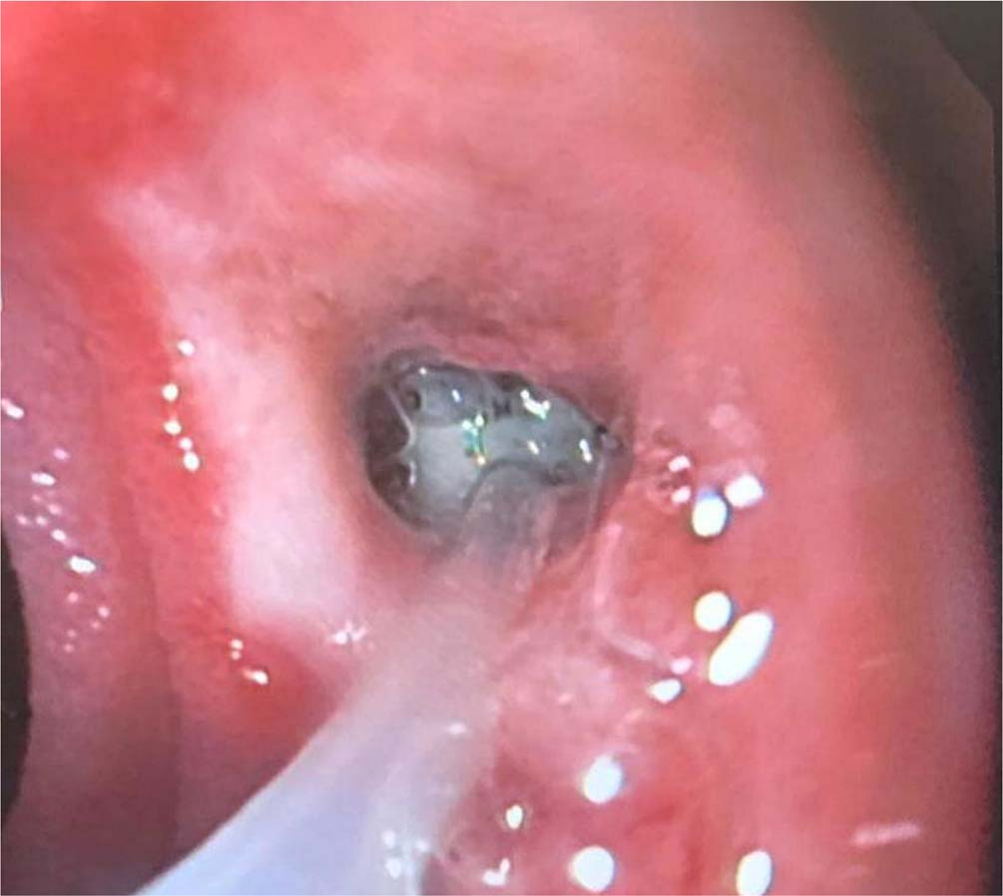
The placement of the catheter and sponge for EVAC therapy is observed.

A 44-year-old female with no significant medical history underwent Roux-en-Y gastric bypass for grade III obesity (BMI 41). She presented with necrosis of the gastric pouch within the first 48 hours, leading to leakage of the gastrojejunal and jejunojejunal anastomoses. Esophagojejunal anastomosis and revision of the jejunojejunal anastomosis were performed, but the patient developed septic shock due to esophagojejunal anastomosis leakage, requiring 7 days in intensive care. Upon stabilization, she was referred to our service for endoscopic management of an esophagojejunal fistula. A 10 mm fistulous orifice was observed (Fig. 1), and EVAC therapy was initiated using a polyurethane sponge with an 8 Fr catheter at 125 mmHg continuous suction (Fig. 2). Six changes were made at 3–5-day intervals.

**Figure 3:**
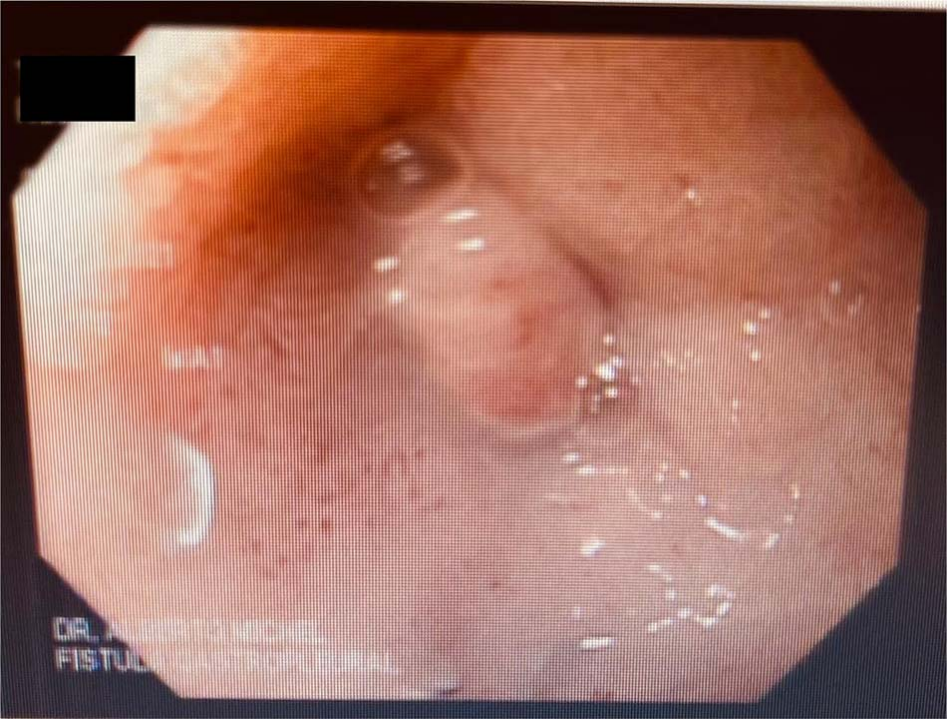
After follow-ups and sponge replacements, the closure of the fistulous defect can be observed.

At the last endoscopy 30 days later, 100% granulation tissue was observed with no evidence of a fistulous orifice (Fig. 3). A leak test by fluoroscopy showed no leakage, allowing the initiation of a liquid diet for 15 days, progressing to a normal diet after one month. Follow-up endoscopy showed no pathological findings.

### Patient B

**Figure 4:**
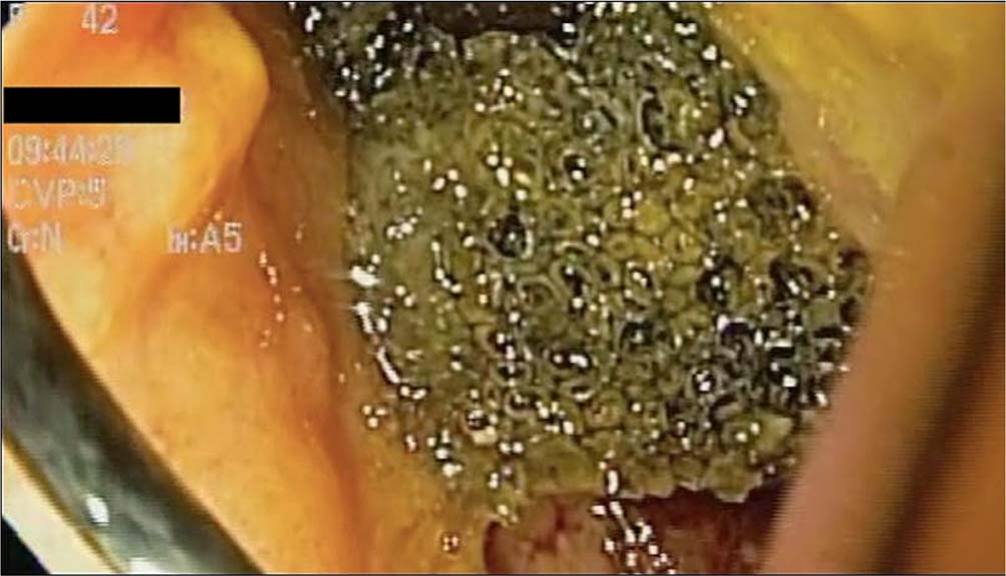
A cavity with an EVAC sponge is seen during endoscopic treatment.

A 41-year-old female with a history of arterial hypertension underwent vertical sleeve gastrectomy for super obesity (BMI 52). She developed a complication of leakage in the cardia region, resulting in a gastropleural fistula and significant bacterial colonization, necessitating a left lobectomy. Persistent cough led to her referral to our department, where endoscopy revealed a 15-mm fistulous orifice. Previous endoscopic management with pigtails and endoscopic glue had failed, prompting initiation of EVAC therapy. Changes were made every 3–5 days, showing partial improvement. A treatment variant was applied by placing the sponge without suction (Fig. 4), promoting granulation tissue formation and enabling the patient to continue oral intake without lung leakage risk.

**Figure 5:**
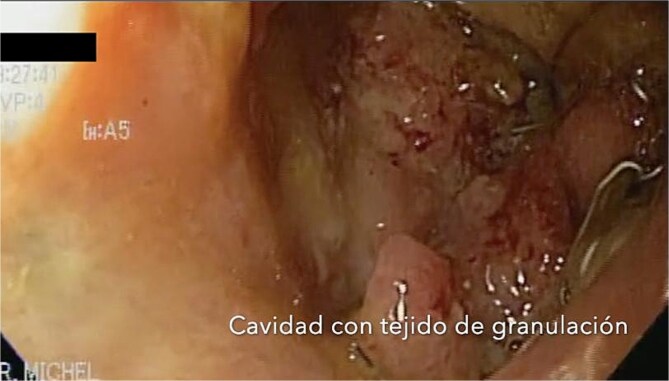
After sponge replacements, granulation tissue is observed in the area where the fistula was located.

This variant, leaving the sponge without a nasogastric tube and negative suction, aimed to manage the fistula while stimulating tissue granulation. Currently, although the fistula persists, she is tolerating oral intake with symptom improvement, and granulation tissue is present, suggesting that the fistula is in the process of closing (Fig. 5).

### Patient C

**Figure 6:**
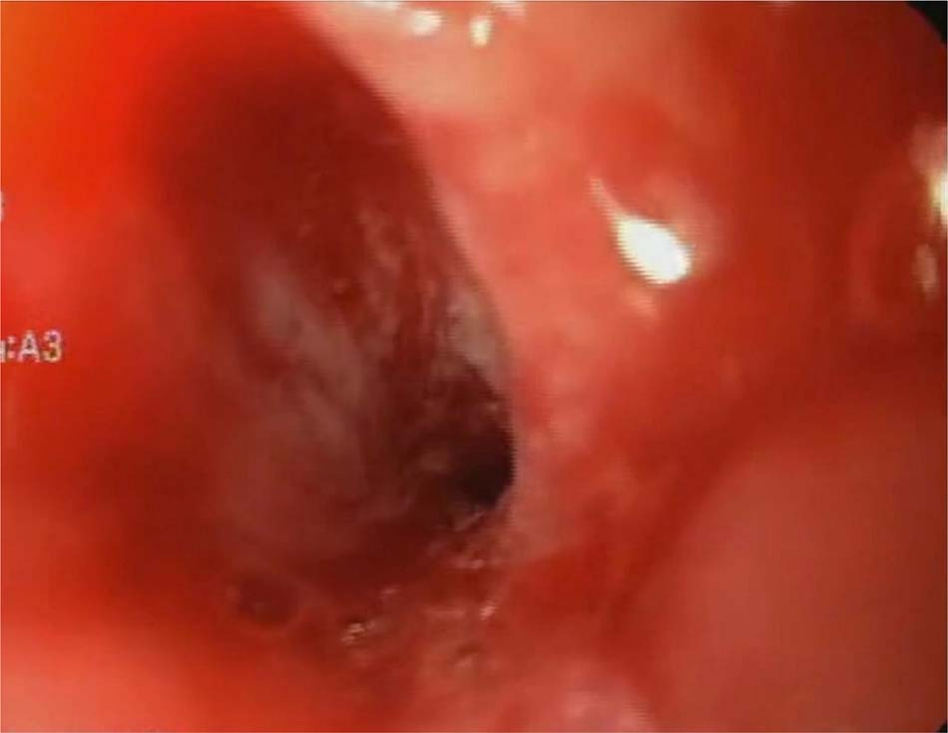
Initial endoscopy shows a fistulous defect below the gastroesophageal junction.

**Figure 7:**
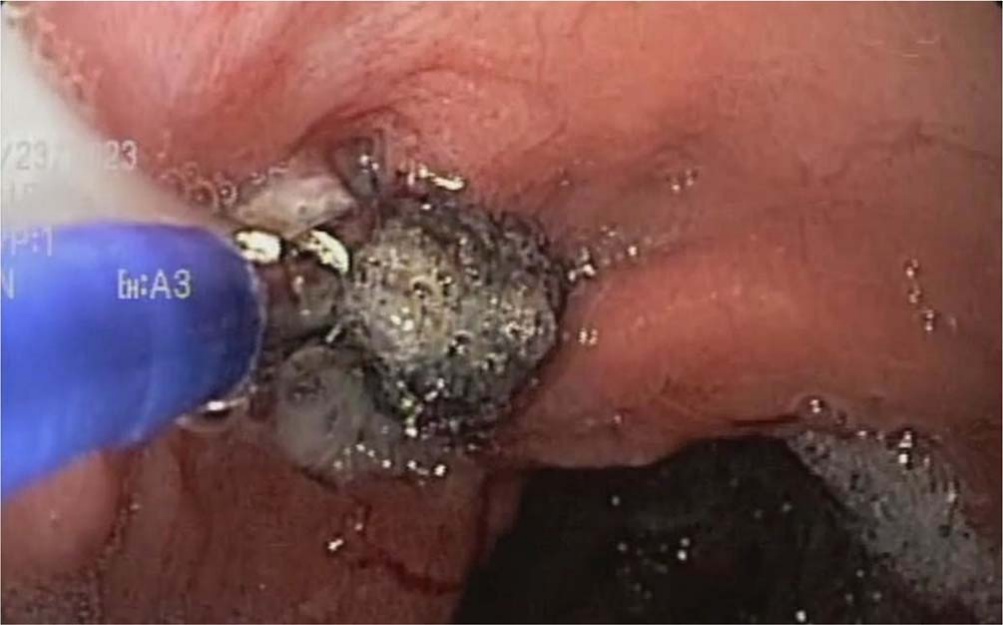
A sponge replacement is performed, revealing a fistulous opening with granulation tissue.

**Figure 8:**
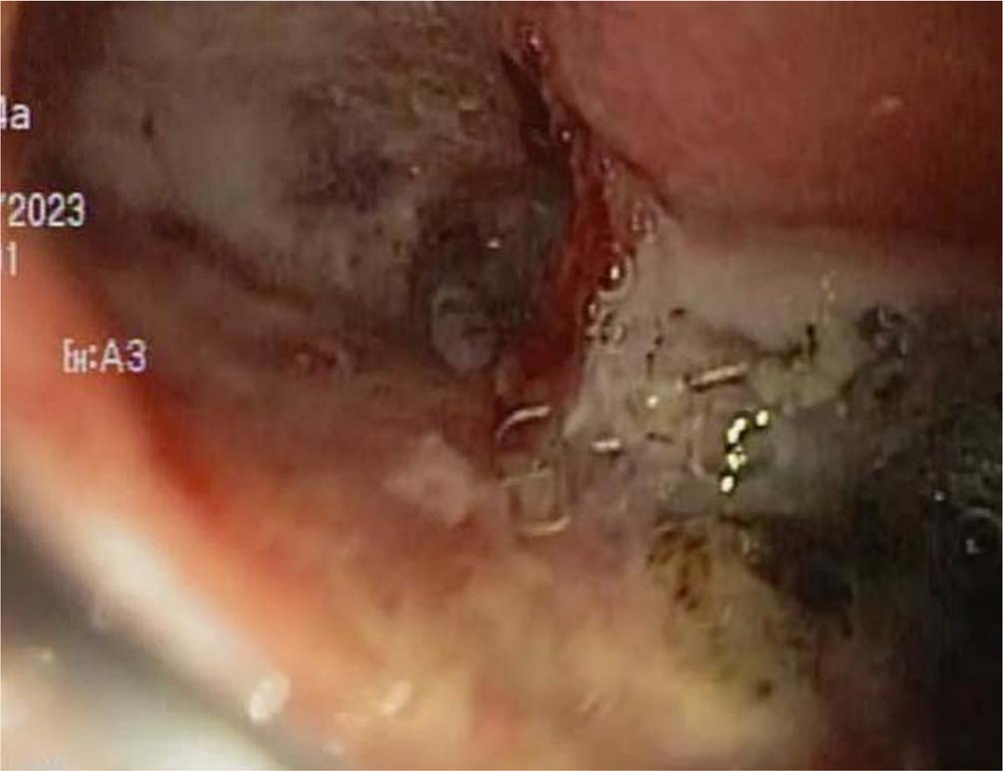
Upon removing the EVAC sponge, we can observe a clean cavernous cavity with minimal fibrin and granulation tissue.

A 48-year-old female without comorbidities underwent vertical sleeve gastrectomy for grade II obesity, developing a leak in the upper third of the sleeve, presenting with a productive cough. Endoscopy revealed a 15 mm fistula (Fig. 6). EVAC therapy was initiated with changes every 3–5 days (Fig. 7). The patient showed slow evolution, developing an intragastric abscess and productive cough. After nine changes over 45 days, endoscopy showed complete resolution of the fistula (Fig. 8).

## Discussion

Endoscopic vacuum-assisted closure (EVAC) has emerged as a promising technique for the management of complex fistulas, particularly those secondary to bariatric procedures. The effectiveness of EVAC in promoting fistula closure is attributed to its mechanism of applying continuous and controlled negative pressure, which facilitates wound drainage, reduces edema, and inhibits microbial growth. These effects collectively enhance granulation tissue formation and subsequent fistula healing [1].

In our series of cases, the successful application of EVAC therapy underscores its potential as a valuable tool in managing challenging post-bariatric fistulas. Patient A, with an esophagojejunal fistula, and Patient B, with a gastropleural fistula, both demonstrated significant clinical improvement following EVAC therapy. The ability of EVAC to manage such complex cases, including those where previous treatments have failed, highlights its efficacy and versatility [3].

Despite the positive outcomes observed, EVAC therapy is not without challenges. The placement of the sponge can be technically demanding, especially in cases with significant fistula angulation, as seen in Patient C. However, even in such complex scenarios, EVAC provided a pathway to successful fistula resolution. This reinforces the importance of technical expertise and careful patient selection in optimizing outcomes with EVAC [8].

Current literature supports the use of EVAC as an effective method for treating gastrointestinal leaks and fistulas. Studies have shown that EVAC is associated with high rates of fistula closure and low complication rates, making it a preferable option compared to traditional methods such as stenting or surgical revision [9]. However, the need for multiple endoscopic sessions and prolonged hospital stays should be considered when planning EVAC therapy [6].

Furthermore, the development of granulation tissue, as observed in our cases, plays a crucial role in the healing process. Granulation tissue formation indicates active healing and tissue regeneration, which are essential for the successful closure of fistulas. This aspect of EVAC therapy not only addresses the immediate closure of the fistula but also contributes to long-term healing and prevention of recurrence [10].

The limitations of EVAC therapy include the technical complexity of the procedure and the requirement for specialized equipment and expertise. Additionally, patient compliance and the ability to undergo multiple endoscopic sessions are critical factors that influence the success of the treatment. Future research should focus on refining the EVAC technique, exploring its applicability in different types of fistulas, and establishing standardized protocols to optimize its benefits [10].

## Conclusion

Even with the closure of complex fistulas using EVAC, the need for vigilant monitoring and early intervention remains critical. This approach ensures the timely identification and management of any complications, thereby improving patient outcomes following bariatric surgery. EVAC represents a significant advancement in the management of post-bariatric fistulas, offering a reliable and effective treatment option that can enhance patient recovery and quality of life.

## Data Availability

The data supporting this study are not publicly available due to confidentiality reasons. However, they can be accessed upon reasonable request and subject to approval by the relevant committees.
